# The effect of exercise-oriented training on physical activity level and exercise awareness in overweight and obese women: A randomized-controlled trial

**DOI:** 10.1016/j.heliyon.2024.e29569

**Published:** 2024-04-24

**Authors:** Zehranur Kacar, Yasemin Cayir, Mahcube Cubukcu, Esra Cinar Tanriverdi

**Affiliations:** a Çekerek Health Center, Yozgat, Turkey; b Department of Family Medicine, Ataturk University Faculty of Medicine, Erzurum, Turkey; c Department of Family Medicine, Samsun University Faculty of Medicine, Samsun, Turkey; d Department of Medical Education, Ataturk University Faculty of Medicine, Erzurum, Turkey

## 1 Introduction

Overweight and obesity are important health problems that pave the way for many diseases with a rapidly increasing prevalence in Turkey and across the world. Obesity is regarded as a multifactorial chronic disease with adverse effects on health. It is the second most important preventable cause of death after smoking. The World Health Organization (WHO) reported obesity to become the most important health problem of the 21st century [1]. According to the WHO data, 39 % of the total adult population aged 18 and over in the world were overweight and 13 % were obese in 2016 [2].

For the diagnosis of obesity, the most reliable measurement methods are those that can measure the amount of adipose tissue in the body most accurately and directly. However, these methods are not recommended for daily clinical practice due to their difficult use and high cost. Generally, low-cost and easily applicable anthropometric measurements are preferred, such as body mass index (BMI), measurement of skin folds, and waist circumference (WC) and hip circumference [3].

The decrease in the physical activity level together with an irregular and unhealthy diet is one of the most important factors for increasing in the prevalence of obesity among people. With the convenience of technological developments and modern life, machines replaced manual labor in many business lines as a significant factor in reducing the physical activity level of society. Today, people have started to adopt a more sedentary lifestyle since they can handle their daily work with less physical energy and activity [4].

One in four adults in the general population has been reported to be physically inactive [5]. Physical activity reduces the risk of death from many diseases, prolongs life expectancy by an average of two years, and increases the quality of life [6,7]. Today, the insufficient level of knowledge about the importance of physical activity for a healthy lifestyle and the adoption of an increasingly sedentary lifestyle are among the major factors that increase the incidence of obesity and many chronic diseases, as well as the rate of mortality and morbidity in society [8].

The Health Belief Model (HBM) is an assessment method that increases the patients’ awareness and enables them to comprehend their health status more accurately. Many studies show that patients who were informed within the framework of the model changed their attitudes and beliefs about health, became more conscious about their health, adopted a more consistent approach regarding treatments and exercise, and decreased barriers to treatment [6,9].

Raising awareness about obesity, increasing the level of physical activity, and adopting healthy living habits play an important role in the prevention and treatment of obesity. One of the most important elements to combat common health problems in society is structured health education. However, studies on the treatment of obesity mostly focus on the effectiveness of medical nutrition programs, and the comparison of pharmacological treatments or surgical procedures [10,11]. In the literature, there is a limited number of studies on awareness about the effectiveness of physical activity/exercise and exercise-oriented training in obesity management [12]. This study aims to examine the effect of exercise-oriented training on physical activity levels and awareness of exercise among overweight and obese women.

## 2 Materials and methods

### 2.1 Ethical Statements

Before initiating the trial, Ethics Committee approval was obtained from Ataturk University Faculty of Medicine Clinical Research Ethics Committee (B.30.2.ATA.0.01.00/331, number:43) and the study was supported by the Ataturk University BAP commission with the project code TTU-2021-9585. Written informed consent was obtained from the patients who agreed to participate in the study for the publication of any clinical data and other data included in the manuscript. The study was conducted following the principles of the Declaration of Helsinki.

### 2.2 Study design and participants

**Fig. 1:**
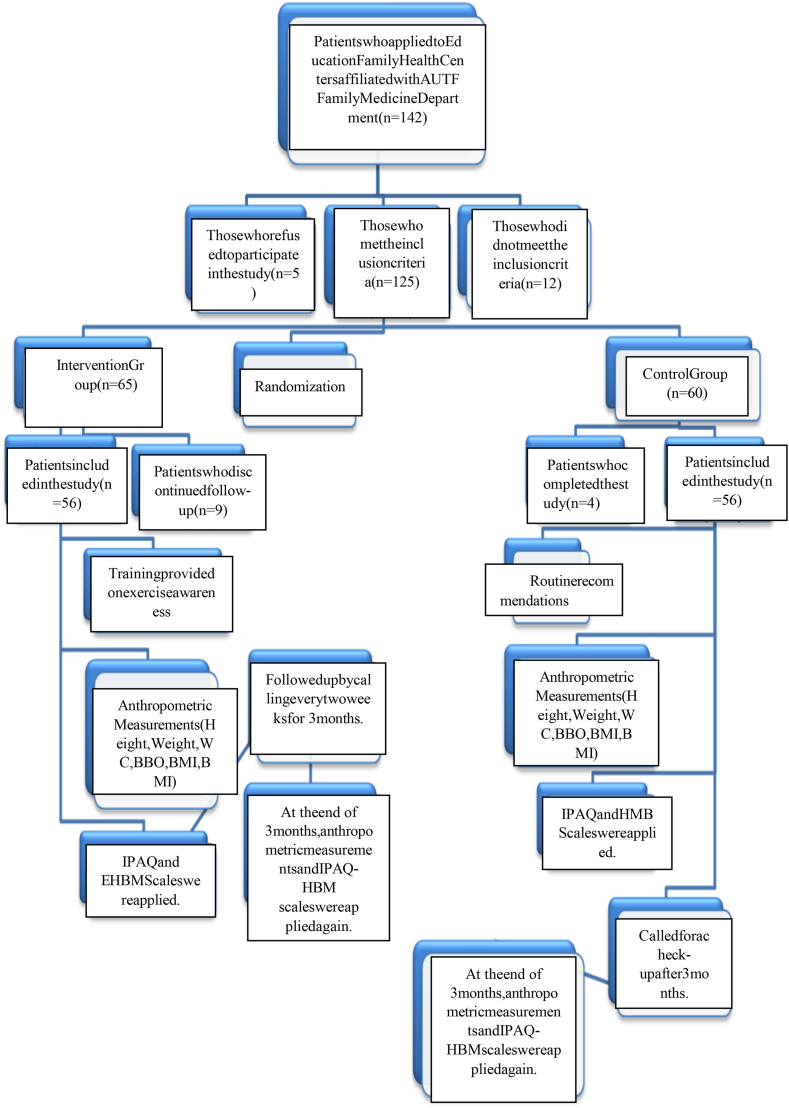
Flow chart of the study.

This study was carried out as a randomized-controlled clinical trial between August-2021 and February-2022. This study is registered on the website of ClinicalTrials.gov (www.clinicaltrials.gov) with the number of NCT06123273. The participants of the study are female patients aged 18–65 years, with a body mass index (BMI) of 25–35 kg/m^2^, who applied to the family health centers of Ataturk University Faculty of Medicine. It was calculated that there should be 56 subjects in each group for 5 % types I error and 80 % power in the sample calculation. 142 people were invited to participate in the study, and it was completed with 112 subjects with 56 participants in each group. The subjects were followed up for three months. The flow chart of the study is given in Fig. 1. Written informed consent was obtained from the subjects who agreed to participate in the study.

The patients were divided into two groups as the control group (CG) and the intervention group (IG) according to the order of admission to the outpatient clinic by the simple randomization method. Individuals with disabilities to exercise, pregnant/breastfeeding women, and individuals with Stage 2–3 obesity (BMI >35 kg/m^2^) were excluded from the study.

The International Physical Activity Questionnaire Short Form (IPAQ) was used to determine the physical activity level at the beginning and end of the study (at the end of the third month) and the Exercise Health Belief Model Scale (EHBM) was used to evaluate the exercise awareness levels of all participants, and their anthropometric measurements (height, weight, BMI, WC, waist-hip ratio (WHR), and body fat percentage (BFP) were recorded.

The CG received no other interventions except routine recommendations (medical nutrition therapy and 30 min of moderate-intensity exercise 5 days a week). After the first meeting, the IG received an exercise-oriented training with a 20-min presentation including graphics, in line with clinical guidelines [13,14], in addition to routine recommendations. The training included information about the benefits of exercise and the harms of a sedentary life, the relationship between exercise and chronic diseases, suggestions to have a more active lifestyle and on how to exercise, the duration and form of exercise necessary for a healthy life, and the things to be considered while exercising. During the three-month follow-up, the subjects in the IG were called every two weeks to ask about their weight measurements, their level of exercise, and how much they walked. The subjects’ questions, if any, were answered, and the interview was completed by making exercise suggestions.

### 2.3 Data collection tools

#### 2.3.1 International Physical Activity Questionnaire short form (IPAQ)

The IPAQ scale was developed by Craig et al., in 2003 [15]. The Turkish validity and reliability studies of the scale were conducted in 2010. The IPAQ score is a questionnaire that questions the time individuals spend on activities in daily life by recording different levels of physical activity for at least 10 min at a time in one week. The subjects are divided into three categories as inactive, minimally active, and very active [16].

#### 2.3.2 Exercise Health Belief Model Scale (EHBM)

The EHBM scale was developed by Esparzo-Del Villar et al. in Mexico in 2017 to determine the health behaviors of individuals toward exercise [17]. A Turkish validity and reliability study was conducted by Yilmaz, and the cronbach's alpha value of the sub-dimensions of the scale was found to be between 0.85 and 0.93 [18]. The EHBM scale consists of five sub-dimensions and a total of 25 items. Likert-type (5-point) scoring system is used in the evaluation of the EHBM scale. It has been reported that the health belief related to that sub-dimension also increases as the score calculated in the evaluation of the scale increases [17,18].

#### 2.3.3 Anthropometric measurements

Weight, BMI, and total fat ratio (TFR) were measured with the Tanita TBF 300® device performing bioelectrical impedance analysis. Before the measurement, the participants took off their shoes, and the measurements were conducted with thin clothes. The researchers paid attention to ensuring that the subjects did not have metal or gold on them, did not use diuretics, and did not need to urinate. The participants were informed that they should attend each control in the same clothes and measurements were performed in that condition. All measurements were recorded by the same person.

While measuring the height; the heels, hips, and shoulders of the individuals were leaning on the stadiometer. During the measurement, it was observed that the back and shoulders should be upright, the chin should be parallel to the ground and the eyes should be facing straight ahead, the feet should be adjacent and slightly open to the side.

A non-flexible tape measure was used for WC and hips circumference (HC) measurements. Subjects were asked to stand upright with their feet close to each other on a flat surface and arms at both sides. After the posture of the individuals was adjusted, WC measurements were made over a thin garment from the middle point of the iliac bone and the last rib bone parallel to the ground while the subject was exhaling. The HC measurement was made parallel to the ground at the widest diameter passing over the gluteus maximus posteriorly and the symphysis pubis anteriorly, which is the highest point of the individual's hip [1,12].

#### 2.3.4 Statistical analyses

SPSS 22.0 (IBM, Armonk, NY, USA) statistical package program was used for statistical evaluation of the data. Descriptive statistics were given as mean standard deviation (SD) and n (%). The chi-square test was used to compare categorical data between groups. Student's t-test was used in cases where the numerical data between the two groups showed normal distribution, and the Mann-Whitney *U* test was used in cases of abnormal distribution. ANOVA test was used to compare numerical data between more than two groups. The significance level was accepted as p < 0.05.

## 3 Results

The study was completed with a total of 112 female patients between the ages of 18–65 and BMI between 25 and 35 kg/m^2^, including the IG (n = 56) who were given exercise training and the CG (n = 56), who did not receive any intervention other than routine recommendations. At the end of the study, the data of a total of 112 people were analyzed. The sociodemographic characteristics of the subjects are given in Table 1. No statistically significant difference was found when the sociodemographic characteristics of the groups were compared (p > 0.05).

**Table 1:** Socio-demographic characteristics of participants.

Descriptive characteristics	Intervention group (n = 56)	Control group (n = 56)	P value
**Mean age of participants (years)**	40.0 ± 9.0	38.6 ± 9.7	>0.05
**Marital status** Married Single	92.90 % (n = 52) 7.10 % (n = 4)	82.10 % (n = 46) 17.90 % (n = 10)	>0.05
**Educational status** Literate <8 year 8–12 year >12 year	5.4 % (n = 3) 51.8 % (n = 29) 26.8 % (n = 15) 16.1 % (n = 9)	10.70 % (n = 6) 44.60 % (n = 25) 25 % (n = 14) 19.60 % (n = 11)	>0.05
**Employment status** Employed Non-employed	7.1 % (n = 4) 92.9 % (n = 52)	19.60 % (n = 11) 80.40 % (n = 45)	>0.05
**Menopause status** Menopaused Non-menopaused	19.6 % (n = 11) 80.4 % (n = 45)	23.20 % (n = 13) 76.80 % (n = 43)	>0.05

There was no statistically significant difference between the groups in terms of the average weight measured at the beginning of the study (p > 0.05). There was statistically significant difference between the groups in terms of weight measured at the end of the study (p < 0.05). After three months of follow-up, there was an average of 2.9 kg and 800 g reduction in body weight in IG and CG, respectively. A significant difference was found in terms of weight loss (p < 0.05). While there was a mean decrease of −1.1 ± 4.7 kg/m^2^ in the BMI of the IG throughout the study, there was a decrease of −0.3 ± 4.7 kg/m^2^ in the CG, which revealed a statistically significant difference between the groups in terms of BMI (p < 0.05). Regarding the BMI change during the study, there was a difference of −2.8 ± 2.1 in the IG, while there was a difference of −1.2 ± 2.3 in the CG, and a statistically significant difference was observed between the two groups (p < 0.05). There was no statistically significant difference between the groups at the beginning and end of the study in terms of WC measurement (p > 0.05). At the end of the three-month follow-up, an average of 5.1 cm thinning was observed in the WC in the IG and 1.7 cm in the control group. There was a significant difference between the groups in terms of thinning in WC (p < 0.05) (Table 2).

**Table 2:** Intergroup comparison of participants’ anthropometric measurements.

Variables	Intervention group (n = 56)	Control group (n = 56)	p value
**Height (cm)**	158.6 ± 5.5	158 ± 5.8	0.55
**Weight** (**kg)** Initial (kg) End (kg) Difference (at the end of three months)	79,9 ± 8 76.9 ± 8.3 −2.9 ± 1.8	75.5 ± 8.1 74.6 ± 7.5 −0.8 ± 2.6	**0.00** 0.12 **0.00**
**BMI (kg/m**^**2**^**)** BMI-1* BMI-2* Difference (at the end of three months)	31.6 ± 2.2 30.5 ± 2.2 −1.1 ± 4.7	30.3 ± 2.3 29.9 ± 2.3 −0.3 ± 4.7	**0.00** 0.22 **0.00**
**WC (cm)** Initial End Difference (at the end of three months)	96.9 ± 7.6 91.7 ± 6.7 −5.1 ± 3.6	94.1 ± 8.1 92.3 ± 8.1 −1.7 ± 3.4	0.06 0.66 **0.00**
**WHR** WHR-1* WHR-2*	0.84 ± 0.06 0.84 ± 0.06	0.85 ± 0.06 0.84 ± 0.06	0.70 0.80
**BFP (%)** BFP-1* BFP-2* Difference (at the end of three months)	33.3 ± 3.5 30.5 ± 2.9 −2.8 ± 2.1	33.3 ± 3.7 32.0 ± 3.2 −1.2 ± 2.3	0,95 **0.01** **0.00**

WC, waist circumference; BMI, body mass index; WHR, waist-hip ratio; BFP, body fat percentage 1*, the value obtained at the beginning of the study; 2*, the value obtained at the end of the study.

The mean score of the IPAQ scale at the beginning and end of the study was 474.1 ± 431.4 Met and 604.8 ± 392 Met, respectively. There was no statistically significant difference between the groups in terms of mean baseline IPAQ scores (IPAQ-1) (p > 0.05). At the end of the study, the mean score of the IPAQ scale (IPAQ-2) was found to be significantly higher in the IG than in the CG (p < 0.05). The activity level of participants was classified according to the IPAQ scale. Accordingly, at the beginning of the study, 75.9 % (n = 85) of all participants were in the inactive group and 24.1 % (n = 27) were in the minimally active group. None of the participants were in the very active group. There was no statistically significant difference between the groups in terms of the IPAQ classification at the beginning of the study (p > 0.05). At the end of the study, 55.4 % (n = 62) of all participants were in the inactive group and 44.6 % (n = 50) were in the minimally active group. There was a statistically significant difference between the groups in the IPAQ classification at the end of the study (p < 0.05). According to the IPAQ, while the initial sitting time was similar, it decreased from 373.4 min/day to 334.2 min/day in IG (Table 3).

**Table 3:** Intergroup of comparison of participants’ IPAQ Scores.

Variables	Intervention group (n = 56)	Control group (n = 56)	p value
IPAQ-1* mean score±SD (MET)	476.8 ± 385.1	471.5 ± 476.7	0.94
IPAQ-2* mean score±SD (MET)	800.3 ± 331.9	409.4 ± 349.6	**0.00**
IPAQ-1* activity level classification Inactive Minimal active	%75 (n = 42) %25 (n = 14)	%76.8 (n = 43) %23.2 (n = 13)	0.82
IPAQ-2* activity level classification Inactive Minimal active	%28.6 (n = 16) %71.4 (n = 40)	%82.1 (n = 46) %17.9 (n = 10)	**0.00**
IPAQ-1* sitting time minute/day	373.4 ± 135.9	409.4 ± 134	0.18
IPAQ-2* sitting time minute/day	334.2 ± 114.2	405.7 ± 120.5	**0.00**

IPAQ, International Physical Activity Questionnaire; SD, standard deviation; 1*, evaluation at the beginning of the study; 2*, evaluation at the end of the study.

The mean score of the EHBM scale was 97.8 ± 10 at the beginning of the study and 105 ± 8 at the end of the study among all participants (p > 0.05). The EHBM mean score of the IG increased significantly at the end of the study compared to the CG (p < 0.05). The mean scores of the EHBM scale sub-dimensions of subjects are given in Table 4. There was no significant difference in any of the sub-dimensions at the beginning of the study and there was a significant difference in all other sub-dimensions, except for the 'beliefs about the seriousness of not exercising' sub-dimension at the end of the study (p < 0.05) (Table 4).

**Table 4:** Comparison of EHBM scale between groups.

Variables	Intervention group (n = 56)	Control group (n = 56)	p value
EHBM-1* mean scores EHBM-2* mean scores	98.5 ± 10.3 110.5 ± 3.8	97.1 ± 9.7 99.5 ± 7.4	0.44 **0.00**
ESHM sub-scales items -1* General health value Beliefs about the vulnerability of not exercising. Beliefs about the severity of not exercising. Beliefs that exercising can reduce threats. Beliefs that the benefits exceed the costs of exercising	9.2 ± 2.9 14.7 ± 0.96 28.9 ± 5.8 28 ± 3.9 17.7 ± 2.9	9.4 ± 2.8 14.5 ± 1.7 29.2 ± 5.3 26.8 ± 4.3 16.6 ± 3.1	0.69 0.28 0.76 0.15 0.06
ESHM sub-scales items-2* General health value Beliefs about the vulnerability of not exercising. Beliefs about the severity of not exercising. Beliefs that exercising can reduce threats. Beliefs that the benefits exceed the costs of exercising	12.4 ± 2 15 ± 0.0 34.9 ± 0.13 29.7 ± 1.1 18.5 ± 2.1	10.5 ± 2.2 14.6 ± 1.3 29.9 ± 4.7 27.7 ± 2.8 16.5 ± 2.3	**0.00** 0.66 **0.00** **0.00** **0.00**

EHBM, Exercise Health Belief Model; 1*, evaluation at the beginning of the study, 2*, evaluation at the end of the study.

The exercise status of the participants is given in Table 5. At the beginning of the study, there was no significant difference between the groups regarding the answers to the questions "How would you describe your exercise status?" "How often do you exercise?” and “What exercises do you do?” (p > 0.05). There was no statistically significant difference between the groups in terms of sociodemographic characteristics, IPAQ Classes (inactive-minimal active), and EHBM mean scores at the beginning and at the end of our study (p > 0.05) (Table 6, Table 7).

**Table 5:** Comparison of exercise status, exercise frequency and exercise type between groups.

Variables	Intervention group (n = 56)	Control group (n = 56)	p value
**Exercise status** Regular Irregular	%14.3 (n = 8) %85.7 (n = 48)	%21.4 (n = 12) %78.6 (n = 44)	0.32
**Exercise frequency** Rarely, occasionally, sometimes <3 days 3–6 day/week Everyday None	%58.9 (n = 33) %10.7 (n = 6) %14.3 (n = 8) %7.1 (n = 4) %8.9 (n = 5)	%44.6 (n = 25) %12.5 (n = 7) %23.2 (n = 13) %12.5 (n = 7) %7.1 (n = 4)	0.50
**Exercise type** Walking Sporting activity Other None	%91.1 (n = 51) %0 (n = 0) %1.8 (n = 1) %7.1 (n = 4)	%87.5 (n = 49) %1.8 (n = 1) %5.4 (n = 3) %5.4 (n = 3)	0.53

**Table 6:** Comparison of participants’ sociodemographic characteristics and IPAQ scores.

Variables	IPAQ-1			IPAQ-2		
	Inactive	Minimal active	p value	Inactive	Minimal active	p value
**Marital status** Married Single	%75.5 (n = 74) %78.6 (n = 11)	%24.5 (n = 24) %21.4 (n = 3)	0.8	%55.1 (n = 54) %57.1 (n = 8)	%44.9 (n = 44) %42.9 (n = 6)	0.88
**Menopause status** Yes No	%66.7 (n = 16) %78.4 (n = 69)	%33.3 (n = 8) %21.6 (n = 19)	0.23	%54.2 (n = 13) %55.7 (n = 49)	%45.8 (n = 11) %44.3 (n = 39)	0.89
**Educational status** Literate <8 years 8–12 years >12 year	%88.9 (n = 8) %81.5 (n = 4) %69 (n = 29) %65 (n = 13)	%11.1 (n = 1) %18.5 (n = 10) %31 (n = 9) %35 (n = 7)	0.28	%77.8 (n = 7) %55.6 (n = 30) %48.3 (n = 14) %55 (n = 11)	%22.2 (n = 2) %44.4 (n = 24) %51.7 (n = 15) %45 (n = 9)	0.49
**Employment status** Employed Unemployed	%60 (n = 9) %78.3 (n = 76)	%40 (n = 6) %21.7 (n = 21)	0.12	%60 (n = 9) %54.6 (n = 53)	%40 (n = 6) %45.3 (n = 44)	0.69

IPAQ-1, International Physical Activity Questionnaire evaluation at the beginning of the study, IPAQ-2, International Physical Activity Questionnaire evaluation at the end of the study.

**Table 7:** Comparison of participants’ socio-demographic characteristics and EHBM scores.

Variables	EHBM-1	p value	EHBM-2	p value
**Menopause status** Yes No	97.4 ± 11.4 97.9 ± 9.7	0.81	103.5 ± 9.7 105.4 ± 7.5	0.32
**Educational status** Literate <8 years 8–12 years >12 years	98.5 ± 11.7 97.5 ± 9.7 96.6 ± 11.9 100 ± 7.1	0.78	103.6 ± 8.5 104.8 ± 9.1 105.1 ± 7 106 ± 6.6	0.72
**Marital status** Married Single	97.7 ± 9.9 98.6 ± 11.2	0.75	105.3 ± 8.2 102.7 ± 6.8	0.25
**Employment status** Employed Unemployed	97.3 ± 10.4 101 ± 6.4	0.18	105.1 ± 8.2 104.6 ± 6.9	0.82

EHBM-1, Exercise Health Belief Model evaluation at the beginning of the study; EHBM-2, Exercise Health Belief Model evaluation at the end of the study.

## 4 Discussion

The results of this randomized-controlled trial indicated that the exercise-oriented training provided a significant increase in the physical activity levels of the overweight and obese women. Moreover, the exercise-oriented training had a beneficial impact on exercise awareness. Besides, it contributed to the improvement of anthropometric measurements.

Due to the rising of sedentary lifestyle in society and the increase in the risk of secondary chronic diseases, the WHO European Strategy Action Plan for the Prevention and Control of Noncommunicable Diseases (2013–2020) focuses on the level of physical activity [19]. Many studies show that the physical activity levels of individuals in society decrease, and the majority of people are considered inactive according to the physical activity scale. In a study carried out by Savci et al. (2006) with university students studying in health departments, it was reported that most of the students were minimally active and their physical activity levels were insufficient [20]. In another study conducted by Genc et al. to determine the physical activity level of bank employees, it was reported that the physical activity levels of the participants were insufficient, and the most common activity was walking, in parallel with our study [21]. In another study, it was reported that 48.9 % of the participants working at desk jobs were minimally active, 25.2 % were inactive, and 25.9 % were active [22]. Similarly, in this study, it was observed that 75 % of the participants were inactive and 25 % were minimally active at the beginning. However, the majority of our participants were doing mild physical activity and most of them were walking.

Many studies in the literature, such as the studies mentioned above, show that the physical activity levels of individuals are insufficient for a healthy life. Physical inactivity is a silent epidemic that leads to many chronic diseases today. Also, physical inactivity ranks fourth among the risk factors as a cause of mortality worldwide [14]. Obesity is one of the most important health problems due to decreased physical activity. The prevalence of overweight and obesity has nearly doubled since 1980; approximately one-third of the world's population is now defined as overweight or obese [23]. Various studies have been carried out for the management of obesity. The effect of education on nutrition and exercise-related behaviors was evaluated in a study carried out with 67 women aged 18 years and older and BMI ≥30 in 2018. As a result of the study, when the participants were evaluated two months after the training, a significant increase was observed in physical activity levels [4]. Similarly, Muller et al. found that education on physical activity, healthy nutrition, and watching TV to combat obesity improved nutrition and physical activity status and had positive effects on reducing sedentary life period [24]. In a study carried out with 200 elderly women in Iran in 2015, it was shown that educational intervention had a positive effect on knowledge and behaviors towards physical activity. When evaluated before training, three months after the intervention, and six months after the intervention, it was seen that the training had a direct and increasing effect on physical activity. In addition, this study showed the training indirectly affected the sub-dimensions of EHBM and had the highest effect on perceived sensitivity, perceived disability, self-efficacy, and behavior [25]. In our study, a significant improvement was observed at the end of three months in the physical activity level, health beliefs, and anthropometric measurements (weight, BMI, WC and BFP) of the IG compared to the CG because of exercise-oriented training and close follow-up. Promoting behavioral change to elevate physical activity levels among individuals as a means to combat obesity and sedentary lifestyles holds significant importance for both personal and public health. Enhancing educational initiatives and training in this area can play a pivotal role in fostering enduring lifestyle changes that promote overall health and well-being. Encouraging a sustainable shift toward healthier living through increased focus on this subject matter can prove highly advantageous.

International Diabetes Federation (IDF) defined the diagnostic criteria for metabolic syndrome in 2005, and it is recommended to use of population-specific WC measurement values in the definition of obesity [1]. Healy et al., in their study carried out in Australia in 2008, examined the effect of the number of breaks on metabolic risk markers and every interruption in sedentary time (mobility) was accepted as a break. It was reported that the participants spent most of the hours they did not sleep during the day sedentary/inactive. Those who took the most breaks during the sedentary period were found to be thinner than those who took breaks the least. As a result of the study, it was observed that the total number of breaks during the sedentary time was reduced and positively affected BMI, WC, triglycerides, and plasma glucose, regardless of the total sedentary time [26]. The increase in the number of breaks during sedentary time and the decrease in BMI and WC with the transition to a more active lifestyle are parallel with the results of our study. In our study, there was no significant difference between the sitting times of both groups at the beginning. At the end of the study, the sitting time of the IG was significantly reduced compared to the CG. However, a significant difference was observed in the IG's WC and BMI values compared to the CG at the end of the study. Both studies showed that adopting a more active lifestyle by reducing the immobile time during the day had positive effects on anthropometrics measurements.

In a study carried out by Khodaveisi et al. between 2018 and 2019, 130 faculty members at Hamadan University of Medical Sciences were included in IG and CG, and the IG attended training sessions for three weeks. Before and two months after the training, the CG and IG were evaluated with the demographic information questionnaire, EHBM minute/day and IPAQ scales. In this study, it was observed that exercise training had a significant positive effect on exercise belief; however, there was no significant change in terms of physical activity level [27]. In our study, the mean EHBM score of the IG was significantly higher than that of the CG. Both studies are similar in terms of significant improvement in health beliefs via exercise training. Also, a significant increase was observed in the level of physical activity with education, unlike the study by Khodaveisi et al. in our study. The increase in the level of physical activity also provided an improvement in the anthropometric measurements of participants. We believe that the difference in results is related to questioning anthropometric measurements by calling the participants every two weeks for three months and following them closely.

There are some limitations of this clinical trial. The most important limitation of this study is that only overweight and stage 1 obese people were included, and it was only performed on women. On the other hand, this trial demonstrated the positive influence of exercise-oriented training and close follow-up for improving physical activity, health belief and anthropometric measurements. Further researches are needed to evaluate the effect of training and close follow-up on the level of physical activity in men and in stage 2 or morbid obesity.

## 5 Conclusion

This study evaluated the effect of exercise-oriented training on physical activity levels and beliefs, attitudes, and behaviors about exercise in overweight and obese women. As a secondary output, the effect of exercise training on anthropometric measurements was discussed. Accordingly, the study results show that the training increased the physical activity level of the participants and had positive effects on their exercise health beliefs. Also, a significant improvement was observed anthropometric measurements. It can be concluded that obese and overweight women with insufficient physical activity started to adopt a more active lifestyle and improved their body measurements due to the increase in their physical activity levels thanks to the training that explains the importance of physical activity in addition to routine recommendations without having strict diet programs. Organizing more training and closer follow-up to ensure and maintain lifestyle changes in the fight against obesity and physical inactivity is important for patients to understand the importance of exercise and to be more active.

## Financial support

This study was supported by Ataturk University Scientific Research Projects Fund Office.

## CRediT authorship contribution statement

**Zehranur Kacar:** Methodology, Data curation. **Yasemin Cayir:** Writing – review & editing, Writing – original draft, Conceptualization. **Mahcube Cubukcu:** Writing – review & editing, Supervision. **Esra Cinar Tanriverdi:** Writing – review & editing, Methodology.

## Declaration of competing interest

The authors declare the following financial interests/personal relationships which may be considered as potential competing interests: Yasemin Cayir reports financial support was provided by Ataturk University Faculty of Medicine. Yasemin Cayir reports a relationship with Ataturk University that includes: consulting or advisory and non-financial support. Yasemin Cayir has patent issued to TTU-2021-9585. This study was supported by the Scientific Research Projects Fund of Ataturk University (No: TTU-2021-9585) If there are other authors, they declare that they have no known competing financial interests or personal relationships that could have appeared to influence the work reported in this paper.
